# SURVIVAL AND COMPLICATIONS ASSOCIATED WITH UNCONVENTIONAL ENDOPROSTHESIS RECONSTRUCTIONS FOR PRIMARY BONE TUMORS AND BONE METASTASES

**DOI:** 10.1590/1413-785220243201e283525

**Published:** 2025-04-07

**Authors:** DANIEL CÉSAR SEGUEL REBOLLEDO, RODRIGO DA SILVA CORDEIRO, ANDRÉ MATHIAS BAPTISTA, LUIZ FILIPE MARQUES CORREIA, RICARDO PIETROBON, OLAVO PIRES DE CAMARGO

**Affiliations:** 1 Instituto do Cancer do Estado de São Paulo, São Paulo, SP, Brazil.; 2 Universidade de Sao Paulo, Faculdade de Medicina, Hospital das Clinicas HC-FMUSP, Instituto de Ortopedia e Traumatologia IOT, Sao Paulo, SP, Brazil.; 3 Department of Surgery of Duke University Medical Center, Durham, NC, United States.

**Keywords:** Bone Neoplasms, Joint Prosthesis, Limb Salvage, Neoplasias de Tecido Ósseo, Prótese Articular, Salvamento de Membro

## Abstract

**Objective:**

To evaluate the survival and complications associated with unconventional stent reconstructions for bone and soft tissue tumors.

**Methods:**

We retrospectively extracted data from the ICESP (Instituto do Cancer do Estado de São Paulo) registry system to evaluate postoperative complications, including thrombosis, amputation, infection, tumor recurrence, and death. We also assessed time until complications and reoperations, as well as survival.

**Results:**

We evaluated 108 patients who underwent unconventional stent reconstruction surgeries for bone tumors. There were significant associations between smoking and tumor recurrence, and between type of cancer, patient functionality, hypertension, and the occurrence of postoperative complications, presence of comorbidities, and death after surgery. Patients with soft tissue tumors and those unable to perform normal activities were at risk of earlier complications.

**Conclusion:**

Type of cancer, patient functionality, presence of comorbidities, stent location, and exposure to chemotherapy or radiotherapy are important risk factors associated with complications and survival after unconventional stent reconstruction surgeries. ***Level of Evidence IV, case series, retrospective study.***

## RESUMO

**Objetivo:**

Nosso objetivo foi avaliar a sobrevida e as complicações associadas às reconstruções com endopróteses não convencionais para tumores ósseos e de tecidos moles.

**Métodos:**

Extraímos dados retrospectivos do sistema de registro do ICESP (Instituto do Câncer do Estado de São Paulo). Avaliamos complicações pós operatórias, incluindo trombose, amputação, infecção, recidiva tumoral e óbito. Também avaliamos o tempo até complicações e reoperações, bem como a sobrevida.

**Resultados:**

Avaliamos 108 pacientes submetidos a cirurgias de reconstruções com endopróteses não convencionais para tumores ósseos. Houve associações significativas entre tabagismo e recidiva tumoral, bem como entre tipo tumoral, função do paciente, hipertensão e ocorrência de complicações em pós-operatórias, presença de comorbidades e óbito após a cirurgia. Foi observado que os pacientes com tumores de tecidos moles e aqueles incapazes de exercer atividades normais apresentavam risco de complicações mais cedo.

**Conclusão:**

O tipo de tumor, a função do paciente, a presença de comorbidades, a localização das próteses e a exposição à quimioterapia ou radioterapia são importantes fatores de risco associados a complicações e à sobrevida após cirurgias de reconstruções com endopróteses não convencionais. **Nível de Evidência IV, série de casos, estudo retrospectivo.**

## INTRODUCTION

Bone reconstruction after surgical resection of long bones is a challenging procedure for orthopedic oncology specialists. Reconstruction methods include autografts, allografts, and non-conventional stents, aiming to relieve pain, achieve tumor control, and preserve functionality by restoring the ability to walk or perform usual activities. Ideally, reconstruction should provide immediate stability, preservation and early movement of adjacent joints, and survival throughout the patient’s life.^1^

The use of non-conventional stents in orthopedic oncology represents a significant advancement in treating malignant bone tumors and metastatic lesions. Among the available treatment methods, non-conventional stents have been shown to be a reliable technique that reduces pain, allows early weight bearing and has fewer adverse effects. ^(^ ^2^

Main complications experienced by patients undergoing limb reconstruction with non-conventional stents include surgical complications (infection and healing problems), implant-related complications (wear and loosening, adverse reactions to the material), and specific oncological complications (tumor invasion and bone metastases). ^(^ ^3^

Studies investigating the functional outcome, survival rates, and complications after bone reconstruction with non-conventional stents are essential for improving this surgical treatment technique. To date, the literature on the subject includes retrospective surveys conducted in single centers or with a small sample size. ^(^ ^4^ ^)-(^ ^6^

Our objective was to evaluate the survival and complications associated with reconstructions with non-conventional stents for bone and soft tissue tumors.

## METHODS

### Study design

An observational study was conducted to evaluate the survival and complications associated with reconstructions using non-conventional stents for bone and soft tissue tumors. Its description follows the Strengthening the Reporting of Observational Studies in Epidemiology (STROBE) guidelines. ^(^ ^7^

### Ethics

The Human Research Ethics Committee of the School of Medicine at University of São Paulo approved this study (CEP-FMUSP).

### Context

Data were obtained from the Electronic Health Record system held by ICESP (Cancer Institute of the State of São Paulo, University of São Paulo). All patient record data were stored on the Philips Tasy system server. After organizing the data, regular expression algorithms and human review maximized the amount of information extracted from the unstructured text (e.g., surgical and discharge notes).

### Participants

ICESP patients who underwent surgical procedures for bone and soft tissue tumors involving reconstructions with non-conventional stents were included. Patients with incomplete records and those with a non-tumor diagnosis were excluded.

### Results

Our outcomes of interest included complications after reoperation, including thrombosis, amputation, infection, tumor recurrence, and death. We also assessed time until complications and reoperations, as well as survival.

### Predictors

Predictors included sociodemographic characteristics (age, gender, occupation), weight, height, smoking, alcohol use, stent location, type of cancer, comorbidities, functionality, and whether the patient underwent chemotherapy and radiotherapy.

### Statistical methods

Our exploratory analysis began with a visual exploration of all variables to assess frequency, percentage, and near-zero variance for categorical variables (e.g., gender, occupation, complications, smoking, alcohol use, stent location, type of cancer, comorbidities, functionality, chemotherapy, and radiation therapy). Near-zero variance is found when a categorical variable has a small percentage of a given category, which is addressed by combining different categorizations of variables. We assessed the distribution of numerical variables (including age, weight, height, time until reoperation, and time until complications) and patterns of missing values. ^(^ ^8^ Since time until complication and time until reoperation had a non-normal distribution, they were categorized as 30, 90, 180, and 365 days. Differences between groups were assessed using standardized mean differences (SMD). We considered the following guidelines in interpreting the SMD magnitudes: SMD = 0.2 corresponds to a small effect; SMD = 0.5 corresponds to a medium effect; and SMD = 0.8 corresponds to a large effect. ^(^ ^9^ We also present p-values for t-tests (for numerical variables) and Chi-square tests (for categorical variables). A p-value < 0.05 was adopted as statistically significant.

We applied multiple linear and logistic regression models to assess associations between outcomes and predictors. We report the odds ratio with 95% confidence intervals for categorical variables (e.g., complications) and the categorized time until reoperation and time until complications. Results were statistically significant when the confidence intervals did not exceed the value of 1.0.

Survival models investigated predictors contributing to the duration of complications and survival using Cox proportional hazards models. ^(^ ^1^ We report the hazard ratios with 95% confidence intervals. Results were significant when the confidence intervals did not exceed the value of 1.0. Numbers > 1.0 indicate an increased risk of complications, whereas < 1.0 reduce the risk of complications at a specific time. We also generated Kaplan-Meier survival curves to describe the likelihood of complications after surgical procedures involving non-conventional stents for bone and soft tissue tumors.

Finally, we used regression trees (recursive partitioning) with the same set of results and predictors described above. Regression trees represent the best cut-off points for predictor values in the context of a given variable after considering previous predictors.

## RESULTS

Complications after surgery included reoperation, infection, thrombosis, amputation, tumor recurrence, or death. Our sample had a mean age of 50.7 years (±17.4), mean weight of 68.2 (±11.7), mean height of 168 (±9.56) and mean BMI of 24.3 (±3.86). Most patients were male (56.5%), retired or unemployed (14.8%), nonsmokers (50%), did not consume alcohol (47.2%), and did not undergo chemotherapy or radiotherapy (77.8%). Stents were more frequently placed in the proximal femur (33.3%) and the most common type of cancer was “other” (not in bone, articular, mesothelial cartilage or soft tissues, 66.7%). A total of 36.1% of patients presented at least one comorbidity, 26.9% had hypertension, and 8.33% had diabetes. KPS showed that most patients had normal functionality or mild signs of disease (21.3%). Our results indicate no significant differences between patients who had complications and those who did not.

**Figure 1:**
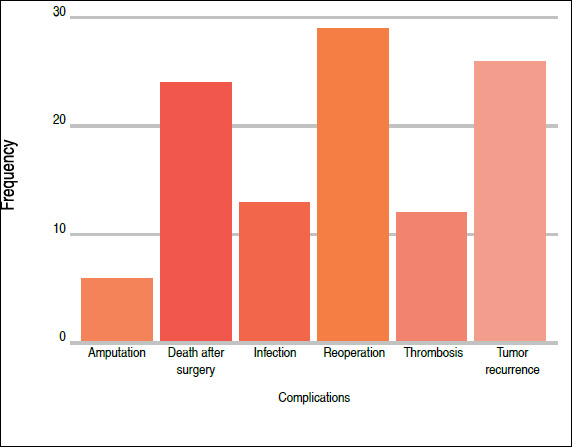
Frequency of complications. Source: prepared by the authors.

Evaluation of the overall frequency of different complications found that a total of 12 (11.1%) patients had thrombosis, 6 (5.56%) underwent amputation, 13 (12%) experienced infections, 26 (24.1%) exhibited tumor recurrence, and 24 (22.22%) died after surgery (Figure 1).

We assessed the association between these outcomes and smoking, alcohol consumption, exposure to chemotherapy or radiotherapy, type of cancer, gender, functionality, and comorbidities. Our results indicated a significant association between nonsmokers and tumor recurrence but found no statistically significant associations between alcohol consumption and death after surgery, thrombosis, infections, tumor recurrence, reoperations at 365 days, and complications at 180 and 265 days.

We found no statistically significant association between gender and death after surgery, thrombosis, amputation, infection, tumor recurrence, complications at 30, 90, 180, or 365 days, and reoperation at 30, 90, 180, or 365 days.

Results indicate no statistically significant associations between exposure to chemotherapy or radiotherapy and death after surgery, thrombosis, infections, tumor recurrence, complications at 30, 90, 180 or 365 days, and reoperations at 30, 90, 180 or 365 days. Given the low percentage of patients who had amputations and underwent chemotherapy or radiotherapy, we were unable to assess the association between exposure to chemo or radiotherapy and this outcome. 

Table 1 presents the odds ratio for the association between type of cancer and each outcome. We found a significant association between mesothelial and soft tissue cancer and complications at 90, 180, and 365 days. Associations between types of cancer and amputation, complications at 30 days, and reoperations at 30, 90, or 180 days were not evaluated due to the low frequency of patients in each of these outcomes who had mesothelial and soft tissue cancer.

**Table 1:** Association between outcomes and types of cancer

	Bone or articular cartilage cancer	Mesothelial soft tissue cancer	Other types of cancer
Death after surgery	1 [Reference]	1.39 (0.036; 37.8) [p = 0.844]	0.549 (0.048; 5.16) [p = 0.603]
Thrombosis	1 [Reference]	5.18 (0.095, 357) [p = 0.397]	0.731 (0.02, 27) [p = 0.852]
Infection	1 [Reference]	4.18 (0.316, 130) [p = 0.313]	0.067 (0.002, 1.15) [p = 0.094]
Tumor recurrence	1 [Reference]	0.968 (0.029; 20.4) [p = 0.984]	0.38 (0.034; 3.54) [p = 0.4]
Complication at 90 days	1 [Reference]	27.7 (1.5; 1,553) [p = 0.047]	3.22 (0.279, 81) [p = 0.383]
Complication at 180 days	1 [Reference]	24.1 (1.26; 1,345) [p = 0.061]	1.42 (0.155; 15.4) [p = 0.756]
Complication at 365 days	1 [Reference]	25.3 (1.2, 1,543) [p = 0.065]	1.78 (0.21; 18.7) [p = 0.602]
Complication after 365 days	1 [Reference]	0.72 (0.029; 9.02) [p = 0.808]	0.207 (0.009; 2.03) [p = 0.219]
Reoperation at 365 days	1 [Reference]	12 (0.564, 591) [p = 0.135]	1.88 (0.045; 70.5) [p = 0.721]

Source: ICESP Archive.

Table 2 presents the odds ratio for the association between functionality and each outcome. Our results indicate a significant association between patients requiring assistance or disability and complications at 180 or 365 days. Associations between functionality and thrombosis, infection, amputation, complications at 30 days, and reoperations at 30, 90, or 180 days were not evaluated due to the low frequency of patients in each of these outcomes with different functionality levels.

**Table 2:** Association between outcomes and functionality

	Normal or minor signs of illness	Normal activity with exertion	Able to take care of oneself, but unable to perform usual activities	Requires occasional assistance	Requires assistance or invalid
Death after surgery	1 [Reference]	2.03 (0.177; 28.3) [p = 0.57]	0.395 (0.013; 7.97) [p = 0.556]	0.521 (0.043; 5.2) [p = 0.581]	0.021 (0.001, 0.319) [p = 0.015]
Tumor recurrence	1 [Reference]	1.33 (0.088; 21.9) [p = 0.834]	0.781 (0.061; 8.78) [p = 0.839]	0.632 (0.057; 5.97) [p = 0.689]	3.08 (0.425; 26.6) [p = 0.276]
Complication at 90 days	1 [Reference]	0.955 (0.062; 15.7) [p = 0.973]	2.24 (0.146; 36.8) [p = 0.552]	0.976 (0.071; 12.9) [p = 0.985]	8.08 (0.877, 123) [p = 0.087]
Complication at 180 days	1 [Reference]	3.13 (0.215; 56.8) [p = 0.409]	6.72 (0.613, 107) [p = 0.136]	3.61 (0.396; 43.2) [p = 0.269]	16.3 (1.79, 257) [p = 0.024]
Complication at 365 days	1 [Reference]	0.647 (0.048; 7.23) [p = 0.726]	4.41 (0.473, 55) [p = 0.208]	3.41 (0.37; 40.6) [p = 0.295]	9.08 (1.01, 129) [p = 0.068]

Source: ICESP Archive.

Table 3 presents the odds ratio for the association between comorbidities and each outcome. Our results indicate a statistically significant association between the presence of comorbidities and death after surgery.

**Table 3:** Association between outcomes and comorbidities

	No comorbidity	Comorbidity
Death after surgery	1 [Reference]	6.4 (1.46; 36.1) [p = 0.02]
Thrombosis	1 [Reference]	1.66 (0.214; 14.6) [p = 0.623]
Amputation	1 [Reference]	1.16 (0.039, 23) [p = 0.92]
Infection	1 [Reference]	0.608 (0.106; 2.94) [p = 0.545]
Tumor recurrence	1 [Reference]	0.783 (0.191; 2.98) [p = 0.723]
Complication at 90 days	1 [Reference]	0.514 (0.117; 2.04) [p = 0.354]
Complication at 180 days	1 [Reference]	0.98 (0.261; 3.61) [p = 0.976]
Complication at 365 days	1 [Reference]	0.64 (0.168; 2.31) [p = 0.499]
Complication after 365 days	1 [Reference]	2.72 (0.636, 13) [p = 0.185]
Reoperation at 30 days	1 [Reference]	4.79 (0.371; 90.7) [p = 0.236]
Reoperation at 90 days	1 [Reference]	4.79 (0.371; 90.7) [p = 0.236]
Reoperation at 180 days	1 [Reference]	2.15 (0.208; 23.1) [p = 0.504]
Reoperation at 365 days	1 [Reference]	2.32 (0.287, 21) [p = 0.424]
Reoperation after 365 days	1 [Reference]	1.76 (0.256; 12.6) [p = 0.558]

Source: ICESP Archive.

Table 4 presents the odds ratio for the association between hypertension and each outcome. Results indicate a significant association between the absence of hypertension and complications after 365 days.

**Table 4:** Association between outcomes and hypertension

	No hypertension	Hypertension
Death after surgery	1 [Reference]	3.15 (0.697; 15.4) [p = 0.138]
Thrombosis	1 [Reference]	1.77 (0.161; 20.9) [p = 0.63]
Amputation	1 [Reference]	0.558 (0.007; 13.2) [p = 0.742]
Infection	1 [Reference]	0.609 (0.073; 3.61) [p = 0.602]
Tumor recurrence	1 [Reference]	2.31 (0.564; 9.83) [p = 0.243]
Complication at 90 days	1 [Reference]	0.39 (0.068; 1.81) [p = 0.247]
Complication at 180 days	1 [Reference]	0.98 (0.237, 4) [p = 0.977]
Complication at 365 days	1 [Reference]	0.725 (0.168; 3.01) [p = 0.656]
Complication after 365 days	1 [Reference]	5.57 (1.05; 38.9) [p = 0.055]
Reoperation at 30 days	1 [Reference]	9.76 (0.566, 382) [p = 0.136]
Reoperation at 90 days	1 [Reference]	9.76 (0.566, 382) [p = 0.136]
Reoperation at 180 days	1 [Reference]	4.65 (0.352; 77.6) [p = 0.236]
Reoperation at 365 days	1 [Reference]	2.04 (0.181; 22.9) [p = 0.538]
Reoperation after 365 days	1 [Reference]	1.62 (0.205; 12.6) [p = 0.633]

Source: ICESP Archive.

### Survival analysis

A Cox proportional hazards model^1^ assessed predictors that contribute to time until complication and survival (time until death). Results of the survival analysis for time until complications indicate that patients with mesothelial soft tissue cancer [6.93 (1.25, 38.5), p = 0.027] and those who were unable to perform normal activities [4.1 (1.6, 10.6), p = 0.003] were at risk of earlier complications, i.e., had higher risk ratios. Conversely, patients who had consumed alcohol in the past [0.191 (0.053; 0.685), p = 0.011] had significantly lower risk ratios (Table 5).

**Table 5:** Time until complications after surgery for reconstruction with non-conventional stents

	Complication
Nonsmoker	1 [Reference]
Former smoker	0.974 (0.296; 3.21) [p = 0.966]
Smoker	0.435 (0.112; 1.7) [p = 0.23]
No alcohol use	1 [Reference]
Past alcohol use	0.191 (0.053; 0.685) [p = 0.011]
Current alcohol use	2.43 (0.456, 13) [p = 0.298]
No chemotherapy or radiation therapy	1 [Reference]
Chemotherapy or radiation therapy	0.452 (0.178; 1.15) [p = 0.095]
Bone and articular cartilage cancer	1 [Reference]
Mesothelial soft tissue cancer	6.93 (1.25; 38.5) [p = 0.027]
Other types of cancer	4.45 (0.892; 22.2) [p = 0.069]
Female	1 [Reference]
Male	0.476 (0.222; 1.02) [p = 0.057]
Able to perform usual activities (KPS)	1 [Reference]
Unable to perform usual activities (KPS)	4.1 (1.6, 10.6) [p = 0.003]
No comorbidity	1 [Reference]
Comorbidity	0.557 (0.273; 1.14) [p = 0.108]
No hypertension	1 [Reference]
Hypertension	0.526 (0.247; 1.12) [p = 0.094]

Source: ICESP Archive.

**Figure 2:**
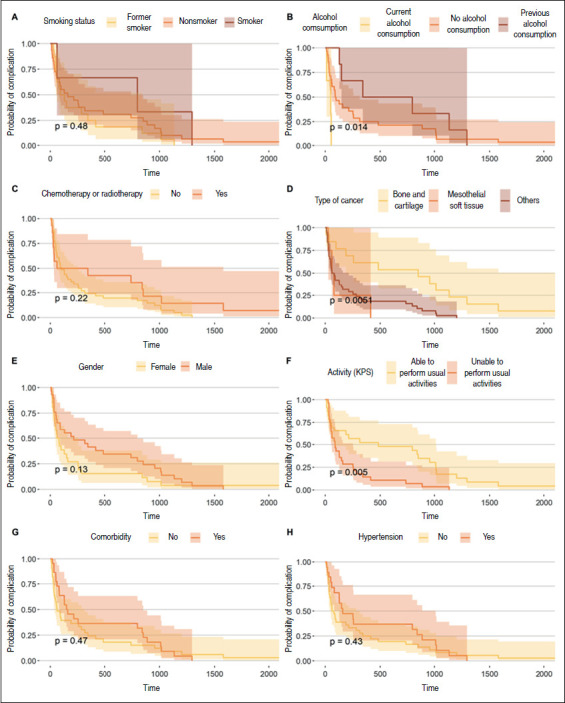
Risk of complications after surgery for reconstructions with non-conventional stents.

Source: prepared by the authors.

Figure 2 illustrates the Kaplan-Meier plots for the risk of early complications after surgery for reconstructions with non-conventional stents. Our results indicate a higher risk of earlier complications for patients who were currently consuming alcohol than for those with past alcohol use (0.014). The analysis also pointed to a higher risk of earlier complications for patients who had mesothelial soft tissue tumors, and those who were unable to perform normal activities. These results corroborate those obtained from the Cox proportional hazards model.

Survival analysis for time until death found that patients who were unable to perform normal activities [12 (1.86; 77.4), p = 0.009] had a risk of earlier death. Patients undergoing chemotherapy or radiotherapy had significantly lower risk rates, i.e., longer survival time (Table 6).

**Table 6:** Time until death (survival) after reconstructive surgery with non-conventional stents

	Time until death
Non-smoker	1 [Reference]
Former smoker	5.57 (0.53; 58.5) [p = 0.152]
Smoker	0.655 (0.048; 9.03) [p = 0.752]
No alcohol use	1 [Reference]
Past alcohol use	0.746 (0.147; 3.79) [p = 0.724]
Current alcohol use	0.484 (0.048; 4.89) [p = 0.538]
No chemotherapy or radiation therapy	1 [Reference]
Chemotherapy or radiation therapy	0.113 (0.015, 0.838) [p = 0.033]
Women	1 [Reference]
Men	0.579 (0.165; 2.02) [p = 0.392]
Able to perform normal activities (KPS)	1 [Reference]
Unable to perform normal activities (KPS)	12 (1.86; 77.4) [p = 0.009]
No comorbidity	1 [Reference]
Comorbidity	3.4 (0.426; 27.1) [p = 0.248]
No hypertension	1 [Reference]
Hypertension	0.595 (0.086; 4.14) [p = 0.6]

Source: ICESP Archive

## DISCUSSION

Limb reconstruction with non-conventional stents has been described in the literature as a safe approach, capable of improving the survival of patients with bone tumors. ^(^ ^9^ As such, studies focused on investigating the prognosis and complications of this type of surgical treatment are increasingly pertinent.

Thomley’s systematic review^10^ highlights a high overall percentage of complications (47%) in stent reconstructions, with an average loss to follow-up of 21% among the included studies. The research reveals that most patients (63%) with reconstructions using primary non-conventional stents have a mean survival of 79 months. Main complications included infections, which required reoperation after 24 months, and tumor recurrence after 36 months postoperative. According to the authors, the loss of follow-up in studies (average of 21%) may underestimate the complication rate, emphasizing the need to create prospective databases to reduce data inconsistency between studies.

Most patients analyzed in our study presented a good functional status, as indicated by KPS, with normal functionality or mild signs of disease in 21.3% of the cases. Survival analysis indicated that patients with mesothelial soft tissue cancer and those unable to perform normal activities were at risk of earlier complications. This highlights the importance of considering the histological type in postoperative planning and prognosis. The association between patient functionality and postoperative complications highlights the need for preoperative functional assessment with at least one validated instrument. 

A large-sample, multicenter study of patients with bone tumors who underwent stent reconstruction of the lower extremity reported significant functional improvement after 1 year, with approximately two-thirds of the sample achieving excellent functionality. Older patients, those with a history of poor preoperative functional outcomes, and patients with soft tissue sarcomas were less likely to report excellent functionality at 1 year. ^(^ ^11^

Analysis of patients’ lifestyle habits, such as smoking and alcohol consumption, showed significant associations with certain outcomes. Our results highlighted a significant association between nonsmokers and tumor recurrence. Despite no statistically significant associations between alcohol consumption and various complications, we observed a significant association between past alcohol consumption and lower complication rates. A higher risk of earlier complications was found for current alcohol users compared with past users.

Tree regression models identified the stent location as the main predictor of complications, emphasizing the importance of appropriately selecting the implantation site of non-conventional stents. Other studies also found a higher frequency of complications in reconstructions with non-conventional stents in the proximal region of the femur. ^(^ ^13^ ^)-(^ ^14^ Chemotherapy or radiotherapy were associated with lower rates of risk of complication, indicating longer survival. This finding differs from that described by Guzik, ^(^ ^14^ who suggests that radiotherapy and preoperative chemotherapy weaken tissues and increase the risk of infections due to tissue damage and necrosis, caused by the former, and the immunosuppression induced by chemotherapy.

One of the limitations in the present study was the retrospective design adopted. We suggest conducting prospective studies with previous sample size calculations and a larger number of participants to validate the present results, since some analyses were limited by sample size.

Due to the short-term follow-up (365 days), results regarding complication rates should be interpreted with caution since many of these potential complications - such as tumor recurrence and denture loosening - can only be observed in the long term. Thus, our results should only guide the prediction of complications after stent grafts in the short term (1 year).

Nonetheless, our study had a significant sample and considered oncological and functionality data of patients with bone and soft tissue tumors. Additionally, the statistical models applied can offer valuable insights for early identification of patients at risk, enabling a more assertive approach to postoperative care and follow-up.

## CONCLUSION

This study provides a comprehensive overview of the complications associated with reconstructions using non-conventional stent grafts in patients with bone or soft tissue tumors, highlighting the importance of considering factors such as cancer, functionality, and stent location for a more personalized approach in managing these cases. Additionally, preoperative functional assessment and a multidisciplinary approach should be prioritized to optimize surgical outcomes and patient survival.
